# A High SNR Improvement CMOS Analog Accumulator with Charge Compensation Technique

**DOI:** 10.3390/s22187050

**Published:** 2022-09-17

**Authors:** Zhongjie Guo, Chen Li, Ruiming Xu, Xinqi Cheng, Changxu Su, Longsheng Wu

**Affiliations:** 1 Department of Electronic Engineering, Xi’an University of Technology, No. 5 Jinhua South Road, Xi’an 710054, China; 2 School of Microelectronics, Xidian University, No. 2 Taibai South Road, Yanta District, Xi’an 710071, China

**Keywords:** TDI CMOS analog accumulator, compensation, charge loss

## Abstract

In this paper, a 7.75 kHz line rate analog domain time delay integration (TDI) CMOS analog accumulator with 128-stage is proposed. An adaptive compensation for the charge loss due to parasitic effects is adopted. Based on the influence mechanism of parasitic effects, alternately charging the top and bottom plates of the storage capacitor while cooperate positive feedback capacitor dynamically compensates for the charge loss of the sampling phase and the holding phase. Using the proposed circuit, after the post-layout simulation verification, the SNR of 128 stage accumulation can be improved by as much as 20.9 dB.

## 1. Introduction

**Figure 1:**
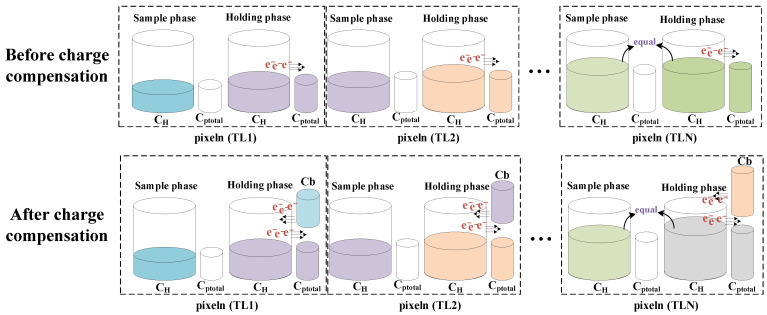
Working diagram of parasitic capacitor effect.

TDI cameras play a key role in the remote push-broom sensing system to improve its low light level capability [1,2,3,4,5,6]. In a TDI image sensor, several stages of pixels are placed in the along-track direction, so TDI image sensor can capture the same target many times in order to extend its equivalent integration time and significantly improve its SNR. In general, the signal is increased by a factor N for an N-stage TDI image sensor, while the uncorrelated noise is increased by the square root of N, the SNR is proportional to the square root of the integration-stage [7,8]. Due to the suppression of noise, TDI camera can be widely used in scanning systems under high scanning speed and low illumination conditions [9]. TDI image sensor is first used in the field of charge coupled devices (CCD), mainly because CCD can easily accumulate charges. However, the accumulation technology based on CMOS technology has not been a good breakthrough [10,11]. The inherent advantages of low power consumption and high integration density of CMOS process have been more and more widely used [12,13]. Unlike CCD, CMOS technology is difficult to solve the problems of on-chip low-noise accumulation and synchronous signal sampling of all pixels in the same column. Therefore, the core problem of TDI technology based on CMOS technology is how to achieve high-precision and high linearity accumulation of low-noise signals. Reference [14] proposed a 25 stages CMOS accumulator based on analog domain, but the hardware consumption of the accumulator is too large, resulting in more area and higher power consumption of the sensor. Reference [15] proposes a 32 stages CMOS accumulator based on analog domain. The sensor realizes high speed and low power consumption. However, with the continuous increase of TDI stage, the parasitic capacitance in the physical layout will significantly affect the effective stages accumulation. high-stage and high SNR improvement have been increasingly demanded. The increase in the number of stages causes the parasitic effects of the storage capacitor network to become more obvious and seriously affect the accumulation effect. As is shown in Figure 1, the total parasitic capacitance C_ptotal_ continuously absorbs the charge in the holding capacitor C_H_ during the charge transfer process. When the charge lost in the holding capacitor C_H_ is equal to the accumulated charge at the time of TLN, the sampling phase of the integrator is equal to the holding phase charge, and the integrator stops the charge accumulate, after charge compensation, the positive feedback capacitor Cb cancels the effect of the total parasitic capacitance.

There are currently two solutions, introducing decoupling capacitors to reduce the impact of parasitic capacitor of storage capacitor network [16], and using two-step accumulation to reduce the number of storage capacitors while introducing decoupling capacitors to reduce the impact of parasitic capacitance [17].129/128 oversampling rates of 128 -stage are added to introduce a total of 25.8 pF capacitors. Two-step accumulation requires a complex control circuit and the area of the capacitor network is not effectively reduced. Use the pipeline charge transfer pixel unit to simulate the CCD charge accumulation method to achieve 45 stage of accumulated charge domain accumulation. Although the line rate has been greatly improved, the charge storage capacity is limited, and the pixel unit is too complex, which limits the expansion of the accumulation series and the accuracy restricted by non-ideal factors [18], the digital domain is combined with the charge domain accumulation. The charge domain accumulation achieves high line rate, and the digital domain realizes the expansion of the accumulation series. The power consumption of this scheme is much greater than other schemes [19], so the analog and digital mixed domain TDI circuit can achieve high-precision accumulation and achieve low power consumption because of reducing the high-frequency clock requirements of the ADC, so this article is dedicated to achieving high-precision analog domain accumulation.

In order to solve the parasitic effects of high-stage digital analog domain accumulation, an analog domain accumulator based on charge compensation technology is proposed. When the circuit is in the sampling phase, the polarity reversal circuit will cause the accumulation in different polarities, the parasitic effects of the two sampling phases before and after cancel each other. In the initial test stage of charge accumulation, the total parasitic capacitance value is determined by the test output result, and the positive feedback capacitance value is quantified by the ADC to configure it to be the same as the total parasitic capacitance values to eliminate the parasitic error in the hold phase. The two charge compensation techniques effectively suppress the parasitic effect of the advanced digital TDI circuit. A 128 stage CMOS TDI analog accumulator is presented in this paper. The second section introduces the mechanism of parasitic effect. The third section shows the schematic diagram of the proposed circuit and experiment results are given in the fourth section, the fifth section presents a brief conclusion.

## 2. Mechanism of Parasitic Effect

**Figure 2:**
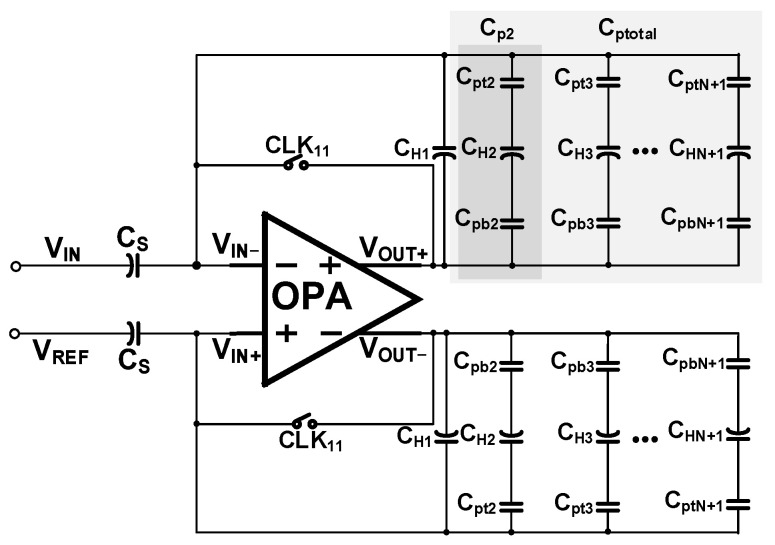
Principle of parasitic effect.

The analog TDI circuit is based on the traditional switched capacitor structure, including three parts: the operational amplifier OPA, sampling capacitor C_S_, and storage capacitor network C_H_. The area of the storage capacitor network will increase with the increasing of accumulation-stage number, therefore, the parasitic capacitor caused between the integrator and the input and output buses cannot be ignored. As is shown in Figure 2, C_pti_ is the parasitic capacitor between the input bus and the bottom plate of the storage capacitor, C_pbi_ the parasitic capacitor between the output bus and the top plate of the storage capacitor. C_pi_ is the equivalent parasitic capacitor of the single-stage integrator, C_ptotal_ is the total parasitic capacitance. C_H_ is the storage capacitor.

**Figure 3:**
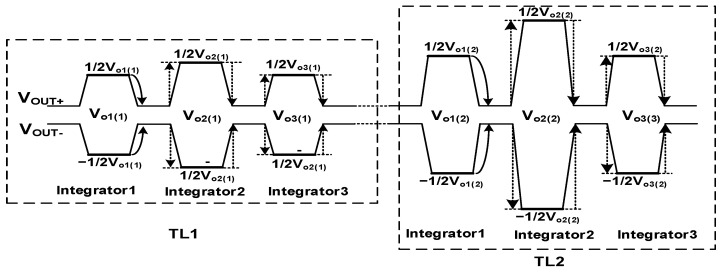
Fully differential OPA differential output waveform diagram.

During the time between TL1 and TL2, the output of OPA is shown in Figure 3. The output of the first integrator in the TL1 stage is V_o1(1)_, and the output in the TL2 stage is V_o1(2)_, which can be found on the output bus. In addition to the accumulation of the voltage change of this stage, for example, the output bus voltage changes caused by the accumulation of the second stage integrator and the third stage integrator are symmetrical and cancel each other, and only the bus voltage change after the accumulation operation of this stage integrator is completed. It will be coupled into the holding capacitor C_H_ every time.

When the circuit works in the sampling phase, parasitic capacitor couples the bus voltage changes into C_H_ and then affects the stored charge. When the circuit works in the holding phase, the total charge is distributed by the charge of the accumulating integrator and the transferring charge. The parasitic effect influence can be formulated as:

(1) $$C_{\mathrm{pi}}=\frac{C_{\mathrm{pti}}C_{\mathrm{pbi}}C_{\mathrm{Hi}}}{C_{\mathrm{pti}}C_{\mathrm{Hi}}+C_{\mathrm{pti}}C_{\mathrm{pbi}}+C_{\mathrm{pbi}}C_{\mathrm{Hi}}}\;$$

(2) $$C_{\mathrm{ptotal}}=\sum_{i=2}^{N+1}C_{\mathrm{pi}}\;$$

(3) $$Q_{\mathrm{in}}=\left(V_{\mathrm{rst}}-V_{\mathrm{sig}}\right)C_{S}\;$$

(4) $$Q_{\left(\mathrm{CH}1\right)o2}=\left[(Q_{\mathrm{in}}\times \frac{C_{H}}{C_{H}+C_{\mathrm{ptotal}}}\times \frac{C_{H}-C_{p}}{C_{H}})+Q_{\mathrm{in}}\right]\times \frac{C_{H}}{{\;C}_{H}+C_{\mathrm{ptotal}}}\;$$

(5) $$Q_{\left(\mathrm{CH}1\right)\mathrm{oN}}=Q_{\mathrm{in}}\sum_{i=1}^{N}{\left(\frac{C_{H}}{C_{H}+C_{\mathrm{ptotal}}}\right)}^{i}{\left(\frac{C_{H}-C_{p}}{C_{H}}\right)}^{i-1}\;$$

In the above equations, Q_in_ is the accumulated charge, Q_(CH1)o2_ is the total charge of C_H1_ in the second accumulation under the holding phase. Q_(CH1)oN_ is the total charge of C_H1_ the Nth accumulation under the holding phase. The formula shows that the value in the accumulation of high-stage numbers cannot be ignored. When the accumulation reaches 128-stage, under the condition of C_H_ and C_S_ is 850 fF, C_ptotal_ is 70 fF and C_p_ of 0.542 fF, the effective accumulation stage can only reach 12.03. When accumulated charge is equal to the parasitic loss charge value, which makes effective accumulation impossible. All current solutions are dedicated to reducing the value of C_p_ to decrease the charge loss. This paper proposes a charge compensation method to offset the charge loss caused by parasitic effect.

## 3. 128-Stage TDI Analog Accumulator with Charge Compensation Technique Circuit Design

**Figure 4:**
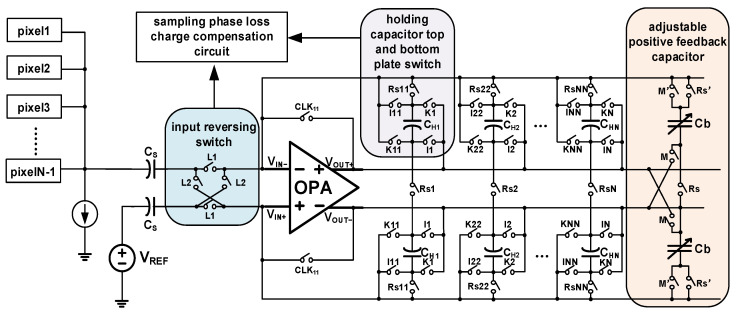
Schematic of proposed circuit.

**Figure 5:**
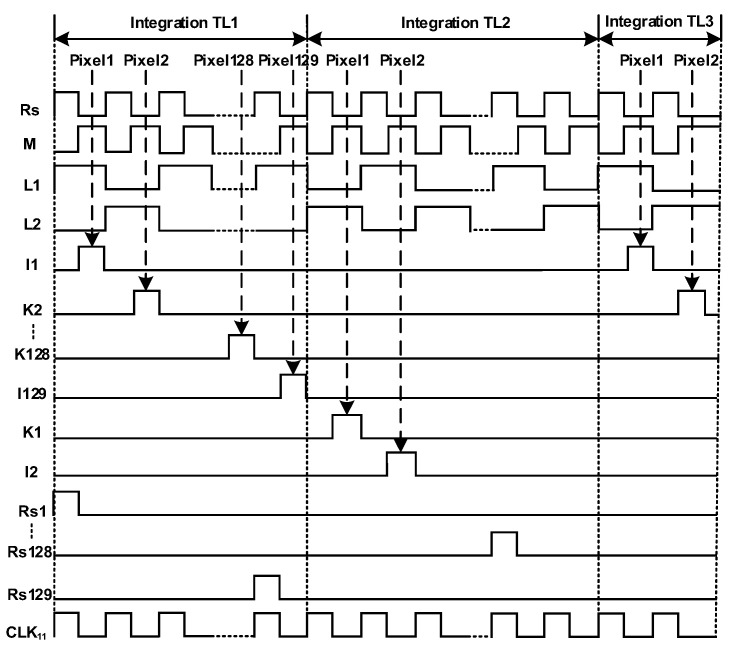
Timing diagram of proposed circuit.

Figure 4 shows a schematic diagram of the proposed circuit; pixel output is accumulated by the analog domain TDI circuit, and after the accumulation is completed, it is output to the subsequent ADC for analog-to-digital conversion. The accumulation circuit is composed of a sampling phase parasitic effect elimination circuit and a maintaining phase parasitic effect elimination circuit and a traditional SC circuit. Figure 5 shows a timing diagram of the circuit control signal. The temporal oversampling technique with oversampling rate of 129/128 is applied in the accumulator. The input signal is read line by line into the corresponding integrator, in the TL1 stage, Rs1 is high when the C_H1_ is the sampling phase and L1 is high, flushing the charge of the integrator, Rs’ high-level flushing the charge of Cb. The CH1 transfer charge through the I1, at the same time, when positive feedback capacitor controlled switch M is a high, Cb provides positive feedback for C_H1_; in the TL2 stage, C_H1_ is in second integration, when L2 is high, under the circumstance of the holding phase, the switch K1 is high, switches the top and bottom plates of the C_H1_ and then transfer charge. Switches Iii, Kii, Rsii, Rs’, M’ are turned off in advance to reduce switch charge injection. The positive feedback capacitor works as the above integration. V_OUT+_ and V_OUT-_ are switched between high and low voltage. The output will be read after the integration times reach 128 times and then repeat the integration process.

### 3.1. Sampling Phase Loss Charge Compensation Circuit Design

Sampling phase loss charge compensation circuit is composed of an input reversing switch and a holding capacitor top and bottom plate switch. As is shown in Figure 3, the charge loss of the sampling phase occurs after the accumulation is completed. The circuit enters the sampling phase from the holding phase, and the output bus voltage is restored from the output voltage to VCM. This voltage change is coupled to the holding capacitor C_H_ through the parasitic capacitance. After 128 times of accumulation, the error is also superimposed 128 times, ignoring the charge loss of maintaining the phase, the charge loss of the sampling phase can be expressed as:

(6) $$Q_{\mathrm{loss}\left(\mathrm{CH}1\right)o2}=Q_{\mathrm{in}}\times \frac{C_{p}}{C_{H}}\;$$

(7) $$Q_{\mathrm{loss}\left(\mathrm{CH}1\right)\mathrm{oN}}=Q_{\mathrm{in}}\sum_{i=1}^{N}{\left(\frac{C_{p}}{C_{H}}\right)}^{i-1}$$

When C_H1_ accumulates and outputs for the first time, its accumulated total charge is Q_in_. After the accumulation is completed, the charge loss due to bus voltage coupling is reflected in the second accumulation as shown in Equation (6). The charge loss and accumulation output had hysteresis. The second accumulation output only reflects the first charge loss. When it is accumulated N times, its charge loss value is shown in Equation (7), and the charge loss increases with the accumulation of the number of stages, the linear increase greatly inhibits the expansion of the accumulation series of the analogaccumulator.

**Figure 6:**
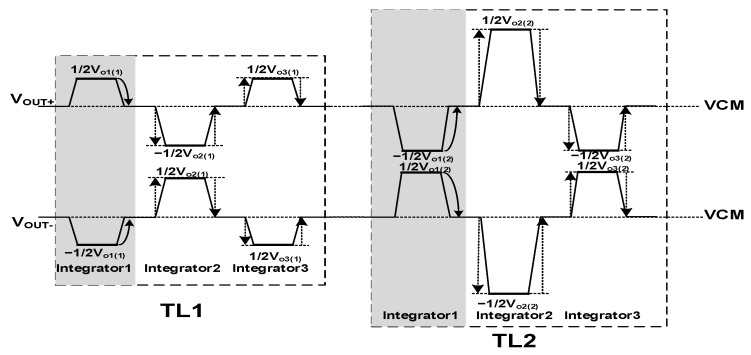
OPA output waveform after output correction.

The single accumulated value is the same, so the output value of each accumulation remains unchanged. Linearity, and the value of the single parasitic effect is small, but in the high-level accumulator, the effect of the parasitic effect is very large after being superimposed for many times. The charge compensation technique proposed in this paper is more suitable for high-stage analog domain accumulators. This paper proposes to measure the voltage of its output bus through the number of stages of output polarity inversion waveform shaping, and showing that the sampling phase charge loss caused by the accumulation output of the previous stage and the subsequent stage cancels each other out, hence improving the accumulation accuracy. Figure 6 shows the output bus voltage after shaping, and Figure 5 shows the timing control of the circuit. In the TL1 stage, there is a dead time in C_H1_. In the TDI circuit between after the holding phase and the next sampling phase, the differential output remains V_o1_. After the sampling phase, CLK_11_ conducts causing its 0 output, and the process is coupled into C_H1_ through parasitic capacitor. In the TL2 stage, L2 is high, and C_H1_ is charged through the bottom plate, so at this time, differential output remains V_o2_, until CLK_11_ conducts in the TL3 stage, which is coupled to C_H1_ to offset the charge loss of C_H1_ last time. When L1 is high, C_Hi_ accumulates the charge through the Ii. When L2 is high, C_Hi_ accumulates the charge through the Ki. Each time the accumulation is performed, L1 and L2 are switched once and the previous parasitic charge effect and the next offsets each other all the time.

**Figure 7:**
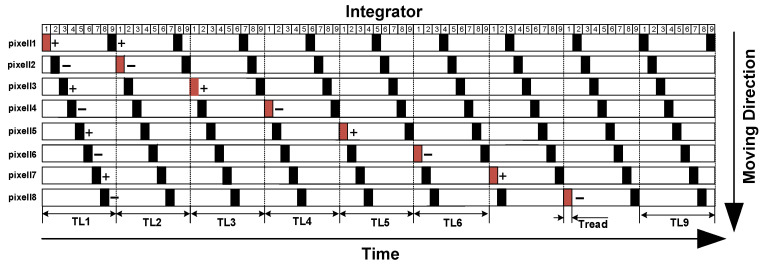
Oversampling rate of 9/8 accumulation process.

The temporal oversampling technique with oversampling rate of 129/128 is applied in the accumulator. Figure 7 shows the charge accumulation process of the over-sampling 9/8 analog accumulator [17]. The output of the first row of pixel units at TL1 is stored in integrator 1, and the output of the second row of pixel units at TL2 is stored in integrator 1. After eight times the accumulation is output to the subsequent ADC at TL8. The polarity when L1 is turned on is positive, and the polarity when L2 is turned on is negative. L1 and L2 are continuously turned on alternately, making the read rectangle shown in Figure 7; the first accumulation output polarity is positive, the second time is negative, the output voltage polarity is always opposite before and after, the first cumulative polarity of each integrator is positive, ensuring the consistency of each integrator output. After sampling the phase charge, the charge loss after compensation can be expressed as:

(8) $$Q_{\mathrm{loss}\left(\mathrm{CH}1\right)\mathrm{oN}}=V_{\left(\mathrm{CH}1\right)o1}\frac{C_{p}}{C_{H}}-V_{\left(\mathrm{CH}1\right)o2}\frac{C_{p}}{C_{H}}+V_{\left(\mathrm{CH}1\right)o3}\frac{C_{p}}{C_{H}}\ldots -{\;V}_{\left(\mathrm{CH}1\right)\mathrm{oN}}\frac{C_{p}}{C_{H}}\;$$

### 3.2. Holding Phase Loss Charge Compensation Circuit Design

**Figure 8:**
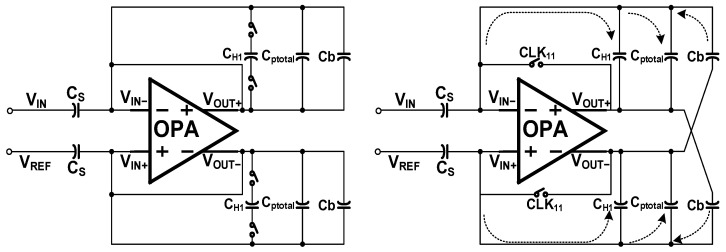
Working principle of positive feedback capacitor.

The charge loss mechanism of maintaining the phase has been studied in depth in the second part. Its parasitic effect can be equivalent to a capacitor connected in series between the input and output buses. This parasitic effect increases the negative feedback coefficient of the loop, so this article proposes to introduce an adjustable positive feedback capacitor to offset this parasitic effect. The input and output characteristics of the fully differential operational amplifier facilitate the introduction of the positive feedback capacitor shown in Figure 8.

**Figure 9:**
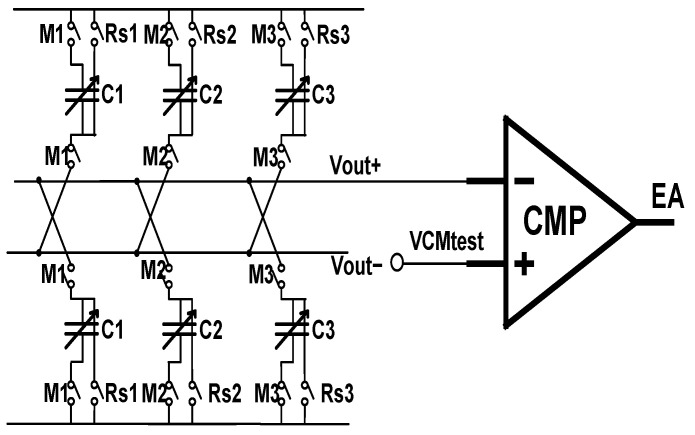
Circuit implementation of the hold-phase parasitic effect cancellation mechanism.

The phase-holding parasitic effect cancellation is achieved by using adjustable positive feedback capacitors. The adjustable positive feedback used in this article is achieved by introducing a test mode, which is implemented in the dummy column in the first column of the CIS array. Only this column in the entire array contains the comparison at the right end in Figure 9. In the test mode, the working principle of the analog domain TDI circuit is exactly the same as that of the traditional CDS switched capacitor circuit, and a step signal of 1 V is input. At this time, the holding phase V_out+_ ideal output value is VCM+500 mV, VCMtest is given by the DAC according to different VCM values. The adjustable capacitor is [0:6] adjustable, and the maximum positive feedback capacitor is 127 fF. When the circuit is in the hold phase, the positive feedback capacitor controls the switch by 1111111-0000000 in descending order. When Vout+ is greater than VCMtest, the EA enable signal is reversed, and the switch signal stops descending. As shown in Figure 9.

The positive feedback capacitor is composed of a control switch and a positive feedback capacitor Cb, and performs a single-stage accumulation. Its value is calculated by the ADC output and the value of Cb is adjusted so that the value of Cb is equal to C_ptotal_. In the sampling phase, Rs’ is high, and the charge in Cb is flushed. In the holding phase, M is high. The voltage difference between V_IN+_ and V_OUT-_, and the value of voltage difference between V_IN+_ and V_OUT+_ are equal and of opposite polarity, which offset the charge distribution of C_ptotal_ to the integrator. The output of the proposed circuit can be formulated as:

(9) $$\begin{array}{l} Q_{\left(C_{H1}\right)o2}=\left[(Q_{\mathrm{in}}\times \frac{C_{H}}{C_{H}+C_{\mathrm{ptotal}}-C_{b}}\times \frac{C_{H}-C_{p}}{C_{H}})+Q_{\mathrm{in}}\right]\\ \times \frac{C_{H}}{C_{H}+C_{\mathrm{ptotal}}-C_{b}} \end{array}$$

(10) $$\begin{array}{l} Q_{\left(\mathrm{CH}1\right)\mathrm{oN}}=\\ Q_{\mathrm{in}}\sum_{i=2}^{N}{\left(\frac{C_{H}}{C_{H}+C_{\mathrm{ptotal}}-C_{b}}\right)}^{i}{\left(\frac{C_{H}-C_{p}}{C_{H}}\right)}^{i-1}{\left(\frac{C_{H}+C_{p}}{C_{H}}\right)}^{i-2}\\ +Q_{\mathrm{in}}\frac{C_{H}}{C_{H}+C_{\mathrm{ptotal}}-C_{b}} \end{array}$$

(11) $$\begin{array}{l} Q_{\left(\mathrm{CH}1\right)\mathrm{oN}}=\\ Q_{\mathrm{in}}\sum_{i=2}^{N}{\left(\frac{C_{H}-C_{p}}{C_{H}}\right)}^{i-1}{\left(\frac{C_{H}+C_{p}}{C_{H}}\right)}^{i-2}+Q_{\mathrm{in}} \end{array}$$

By comparing the above formula (11) and (5), the positive feedback capacitor Cb offsets the parasitic effect of C_ptotal_, and the output of the OPA performs high and low voltage switching so that the parasitic charge loss of the sampling phase offsets each other before and after. The charge compensation type TDI circuit greatly offsets the parasitic and promises the linearity of the output.

## 4. Post-Layout Simulation Results

**Figure 10:**
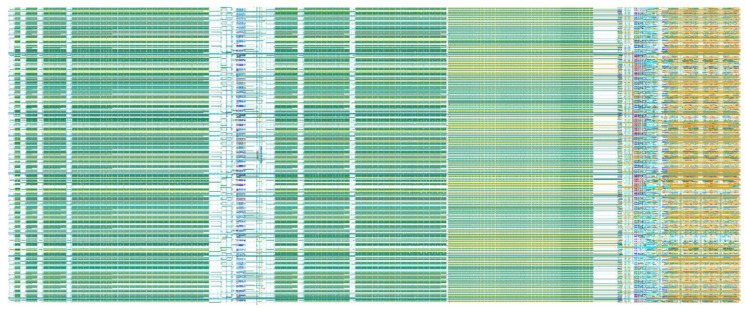
The actual physical implementation layout of the circuit proposed in this paper.

**Figure 11:**
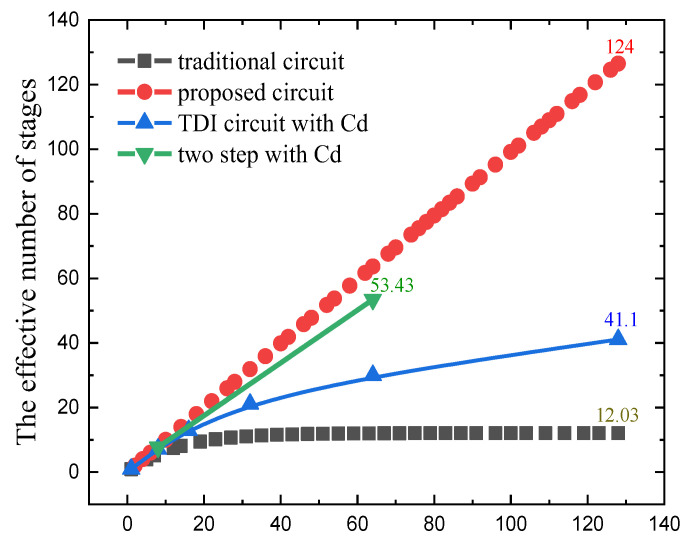
Accumulator input and output curve.

**Figure 12:**
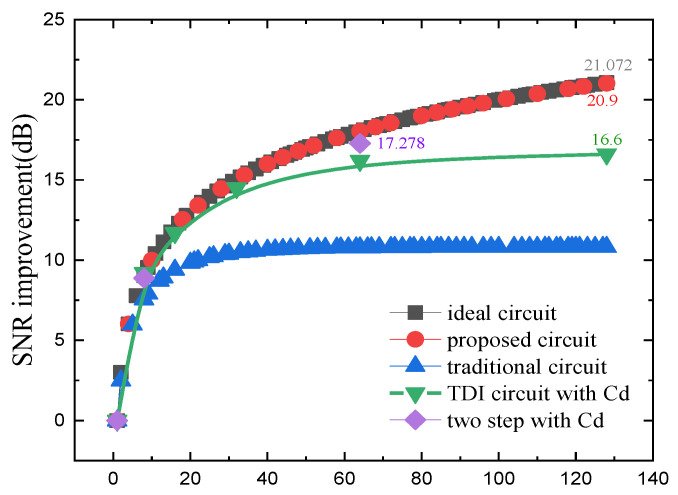
Curves of the SNR improvement versus the designed number of stages.

As shown in Figure 10, the actual physical implementation layout of the circuit proposed in this paper includes column level analog domain accumulation circuit and quantization circuit. The layout area of the 1024 × 128 analog domain is 15.36 mm × 9.535 mm. Figure 11 shows the cumulative output situation in four cases, TDI circuit with Cd [16] and two step with Cd [17] are two existing analog domain accumulation schemes, and this paper designs and verifies the circuit based on 55 nm technology, OPA is gain 84 dB, C_S_ and C_H_ are 850 fF, single integration time is 1 μs, line rate is 7.75 kHz, 128-stage accumulation. The traditional analog accumulator reaches 128-stage of accumulation, and the effective accumulation stages is only 12.03. The effective accumulation of charge compensation TDI circuit reaches at least 124. The effect of signal dependent charge injections and operational amplifier gain errors makes the accumulation impossible to achieve the ideal situation. Figure 12 shows the SNR improvement results of the five methods. The proposed scheme is highly consistent with the ideal curve. In the case of 128-stage accumulation, the average SNR improvement is 20.9 dB, ideally 21.072 dB. The SNR improvement of the previously proposed circuits is less than that of the charge compensation TDI circuit, and it is compared with the state-of-the-art works listed in Table 1.

(12) $$\mathrm{SET}=\frac{△\mathrm{SNR}\times R_{\mathrm{Line}}}{\mathrm{power}}\;$$

where ΔSNR is the SNR improvement of the TDI-CIS, R_Line_ is the line rate; power represents the power consumption of one column pixels, SET [19] is intended to reflect the improvement level of circuit power consumption on line frequency and SNR. The digital domain implementation scheme in Ref [8] has low power consumption and high precision, but the speed is slow, the single accumulation time is 3.25 us. This paper proposes that the circuit single accumulation time is 1 us, so if the parameters in the Ref [8] are converted, the SET value of the circuit proposed in this paper is higher than the scheme proposed in the Ref [8]. Although the line rate of the circuit proposed in this paper is only 7.75 Khz, the SNR improvement effect reaches the lowest 20.9 dB in the case of 128 stage of accumulation, and the SET value of the proposed circuit reaches 558.53 and is better than the hybrid domain TDI circuits. In Ref [19], although the charge and digital hybrid domain accumulation has a line rate of 100 Khz, its power consumption is much greater than other TDI circuits, the charge compensation technology can greatly improve the accumulation accuracy of the analog domain TDI circuit without additional power consumption.

## 5. Conclusions

We present a charge-compensated CMOS-TDI readout circuit for 128-stage accumulation, which dynamically compensates the charge loss caused by parasitic effects. The proposed circuit can improve the SNR by an average of 20.9 dB in 128-stage accumulation. Compared with the previous research, the advantages of the proposed method are: a reasonably high SNR improvement and no large-area decoupling capacitor.

## Figures and Tables

**Table 1:** Performances compared with the state-of-the-art works·.

Specification	This Paper	[8]	[16]	[17]	[19]
**Accumulator**	Analog	Digital	Analog	Analog	Hybrid (Charge, Digital)
**Technology (** ** μm ** **)**	0.055	0.11	0.18	0.18	0.11
**Maximum stage**	128	32	128	64	256
**SNR improvement (dB)**	20.9 @128	14.82 @32	16.6 @128	17.27 @64	24.15 @256
**Line rate**	7.75 k	9.302 k	3.875 k	/	100 k
**SET**	558.53	899.84	131.8	/	231.9
**Power/Column (** ** μW ** **)**	290	153.2	488.3	/	10,413.97

## Data Availability

The data presented in this study are available on request from the corresponding author. The data are not publicly available due to privacy.
